# Intraoperative detection and management of cerebral malperfusion in acute aortic dissection using regional cerebral oxygen saturation monitoring: case report

**DOI:** 10.1186/s40981-026-00853-9

**Published:** 2026-02-10

**Authors:** Shinnosuke Miura, Hiroya Tsujimoto, Ayami Shimomiya, Kenji Yoshitani

**Affiliations:** 1 Department of Anesthesiology, National Cerebral and Cardiovascular Center, Osaka, Japan; 2 Department of Transfusion, National Cerebral and Cardiovascular Center, 6-1 Kishibe-shimmachi, Suita, Osaka, 564-8565 Japan

**Keywords:** Acute aortic dissection, Transesophageal echocardiography, Malperfusion, Carotid duplex ultrasonography

## Abstract

**Background:**

Cerebral malperfusion during acute aortic dissection (AAD) surgery is a life-threatening event requiring prompt detection and intervention. We report a case of intraoperative extension of dissection into the brachiocephalic artery (BCA) detected by regional cerebral oxygen saturation (rSO₂) monitoring before cardiopulmonary bypass.

**Case presentation:**

A 78-year-old man undergoing emergency total aortic arch replacement showed a sudden bilateral rSO₂ decline after anesthesia induction, corresponding to BCA extension on transesophageal echocardiography. rSO₂ recovered during selective cerebral perfusion but fell again during CPB weaning. Carotid duplex ultrasonography revealed collapse of the right common carotid artery due to false lumen expansion compressing the true lumen. Reanastomosis of the BCA restored cerebral oxygenation.

**Conclusion:**

This case highlights the utility of multimodal monitoring—rSO₂ trends, transesophageal echocardiography, and carotid duplex ultrasonography—for detecting and managing cerebral malperfusion during AAD surgery. Early identification allows timely surgical revision and may improve neurological outcomes.

## Background

Cerebral malperfusion in acute aortic dissection (AAD) is a serious complication strongly associated with poor neurological outcomes. Intraoperatively, extension of dissection into the brachiocephalic artery (BCA) can result in an abrupt decrease in cerebral perfusion pressure. BCA dissection has been independently associated with an increased risk of stroke, regardless of the presence of left common carotid artery dissection (odds ratio 3.89) [1]. Regional cerebral oxygen saturation (rSO₂) monitoring is a valuable real-time modality for detecting compromised cerebral perfusion. Although cervical duplex ultrasonography is a standard modality for evaluating cervical arterial flow when transesophageal echocardiography is insufficient, its intraoperative use to confirm dynamic extension of acute aortic dissection into the cervical arteries prompted by real-time rSO₂ changes has not been previously described.

Here, we describe a case in which rSO₂ monitoring detected intraoperative extension of the dissection into the BCA prior to the initiation of cardiopulmonary bypass (CPB). Furthermore, following CPB weaning, expansion of the false lumen in the repaired BCA led to impaired cerebral perfusion, ultimately necessitating a reoperation. Informed written consent was obtained from the patient for publication.

## Case presentation

**Fig. 1:**
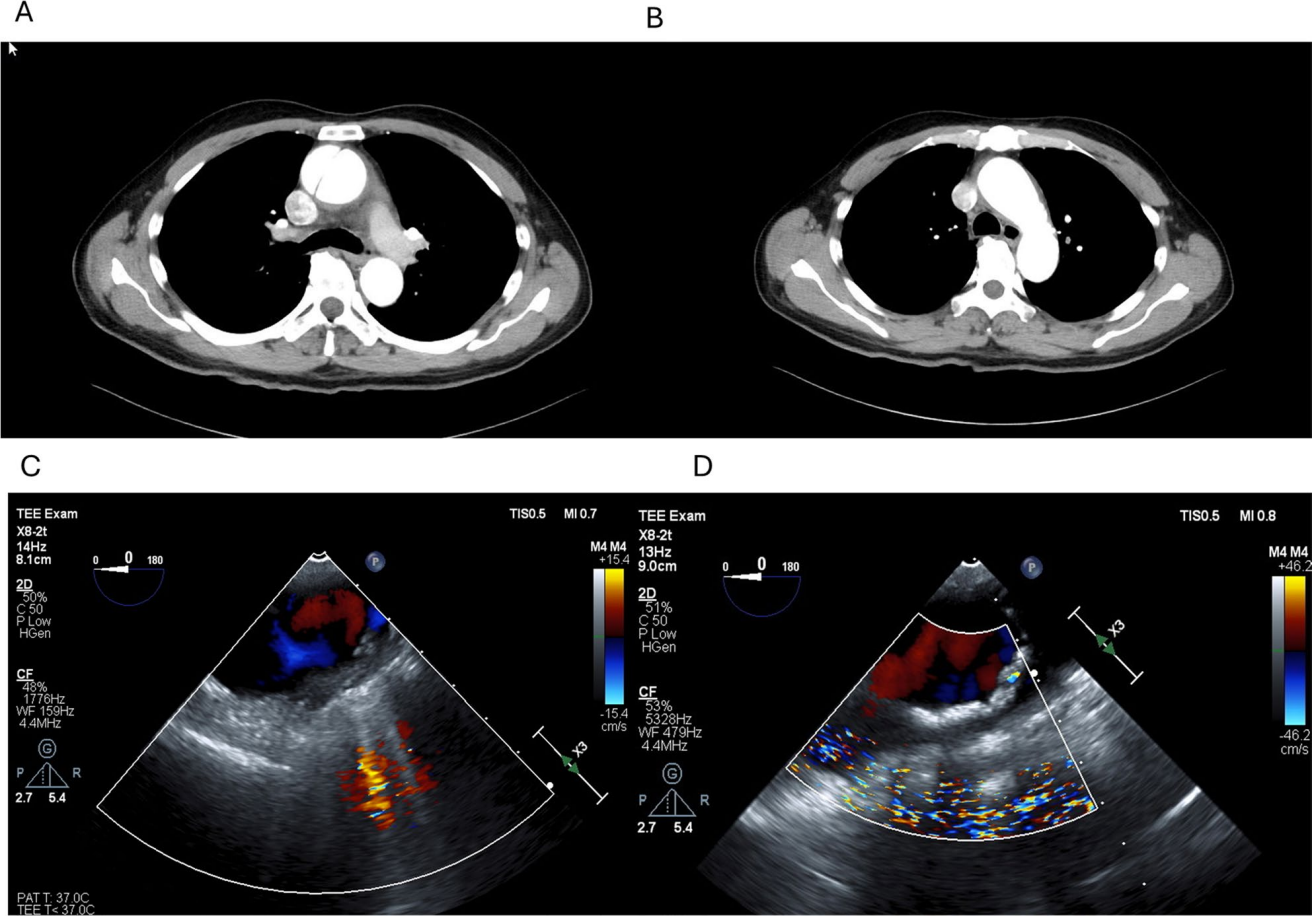
Panels **A** and **B** show preoperative contrast-enhanced chest computed tomography images. A patent false lumen is visible in panel **A**, whereas no dissection is observed in panel **B**, indicating DeBakey type I dissection. Panel **C** shows a transesophageal echocardiography (TEE) image of the aortic arch obtained immediately after anesthesia induction, showing no evidence of a false lumen. Panel **D** shows a TEE image captured at the time of rSO₂ decline, demonstrating the extension of the dissection

A 78-year-old man presented with sudden-onset chest and back pain and was diagnosed with AAD. Emergency total aortic arch replacement (TAR) was performed. Preoperative transthoracic echocardiography revealed moderate pericardial effusion and moderate aortic valve regurgitation. Contrast-enhanced chest computed tomography revealed a dissecting aneurysm of the ascending aorta measuring 60 mm in diameter, consistent with DeBakey type II AAD with a patent false lumen. The dissection was confined to the ascending aorta (Fig. 1A–C) by transesophageal echocardiography (TEE).

The patient was hemodynamically stable, fully conscious, and exhibited no neurological deficit. General anesthesia was induced using total intravenous anesthesia with propofol and remifentanil. Hemodynamic monitoring included left radial artery pressure, right superficial temporal artery pressure (for monitoring the cerebral perfusion pressure during selective cerebral perfusion (SCP)), left dorsalis pedis artery pressure, central venous pressure, and pulmonary artery pressure. Because we use right axillary artery cannulation with brachiocephalic clamping, right radial pressure is not reliable. Thus, right superficial temporal artery pressure is monitored as a surrogate of right carotid perfusion. Transesophageal echocardiography (TEE) was performed to examine the extent of the aortic dissection. Neuromonitoring included SedLine^®^ and O3^®^, and rSO₂ monitoring (Masimo Corp., Irvine, CA, USA) was applied to the forehead.

**Fig. 2:**
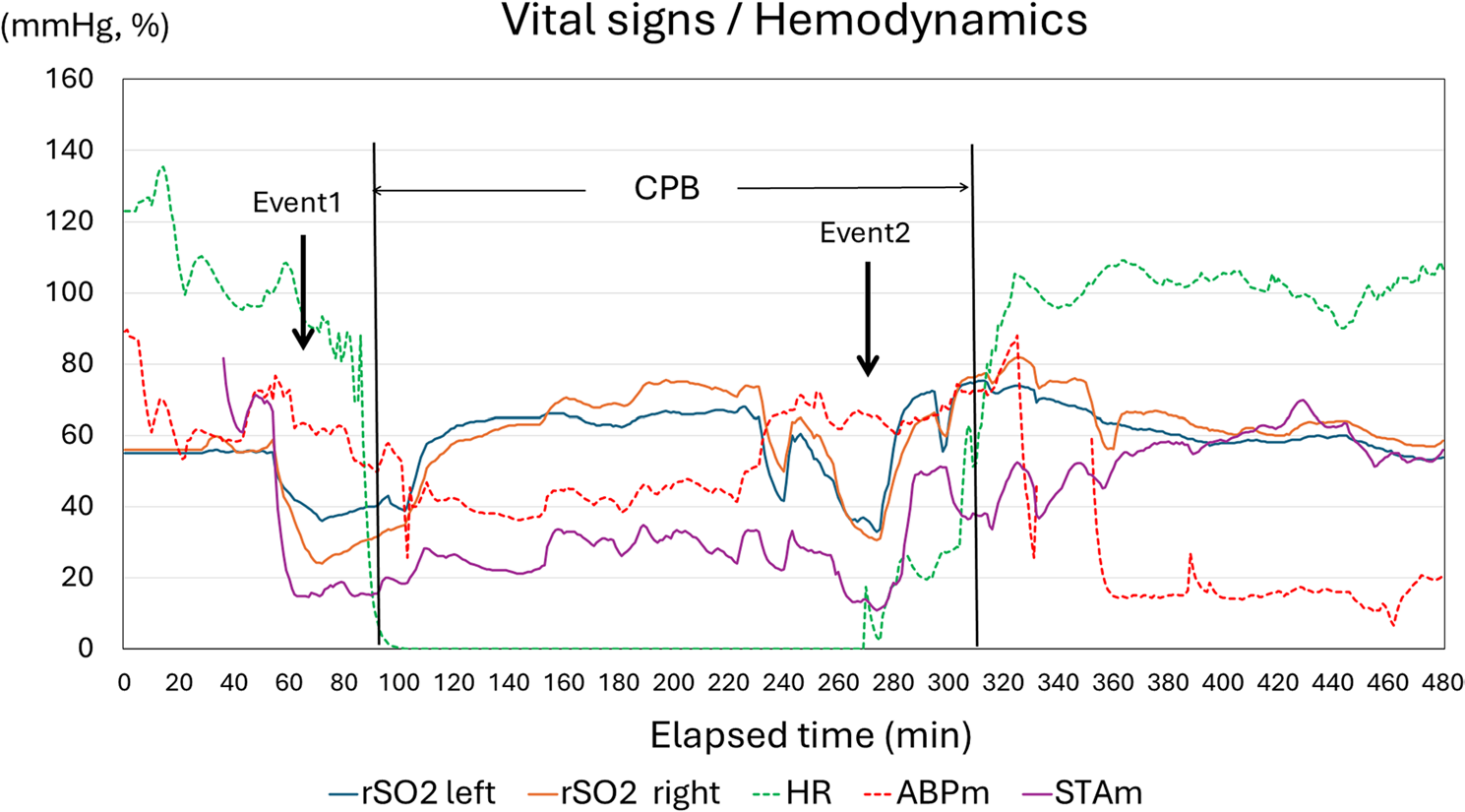
Trend of vital signs. Event 1 indicates a decrease in rSO₂ prior to the initiation of cardiopulmonary bypass (CPB), whereas Event 2 indicates a decrease just before weaning from CPB. ABPm: mean arterial pressure of radial artery: HR: heart rate; STAm: mean pressure of right superficial temporal artery

Baseline rSO₂ values after induction were 56% (left) and 58% (right). Shortly thereafter, the rSO₂ values abruptly decreased to 38% on the left and 25% on the right and superficial temporal artery pressure also decreased (Fig. 2, Event 1). TEE revealed the extension of the dissection into the aortic arch (Fig. 1D). There is a possibility that the dissection flap was compressing the true lumen. These findings were promptly communicated to the surgical team, and CPB was initiated.

The dissection extended into the aortic arch and descending aorta, likely causing malperfusion of the cervical branches and the observed drop in rSO₂. The dissection progressed to DeBakey type I. CPB was initiated via the left femoral artery for arterial inflow and both the superior and inferior venae cavae for venous drainage. The nasopharyngeal temperature was lowered to 25 °C for cerebral protection, followed by the establishment of SCP.

Following CPB initiation, the rSO₂ values improved to approximately 60% bilaterally. Retrograde perfusion from femoral artery cannulation likely augmented contralateral blood flow through the left subclavian and left internal carotid arteries. The SCP was delivered via direct cannulation of the cervical vessels, with a total flow maintained at 800–900 mL/min and left common carotid artery (LCCA) pressure (measured at the tip of the selective cerebral perfusion cannula) maintained at approximately 40 mmHg.

**Fig. 3:**
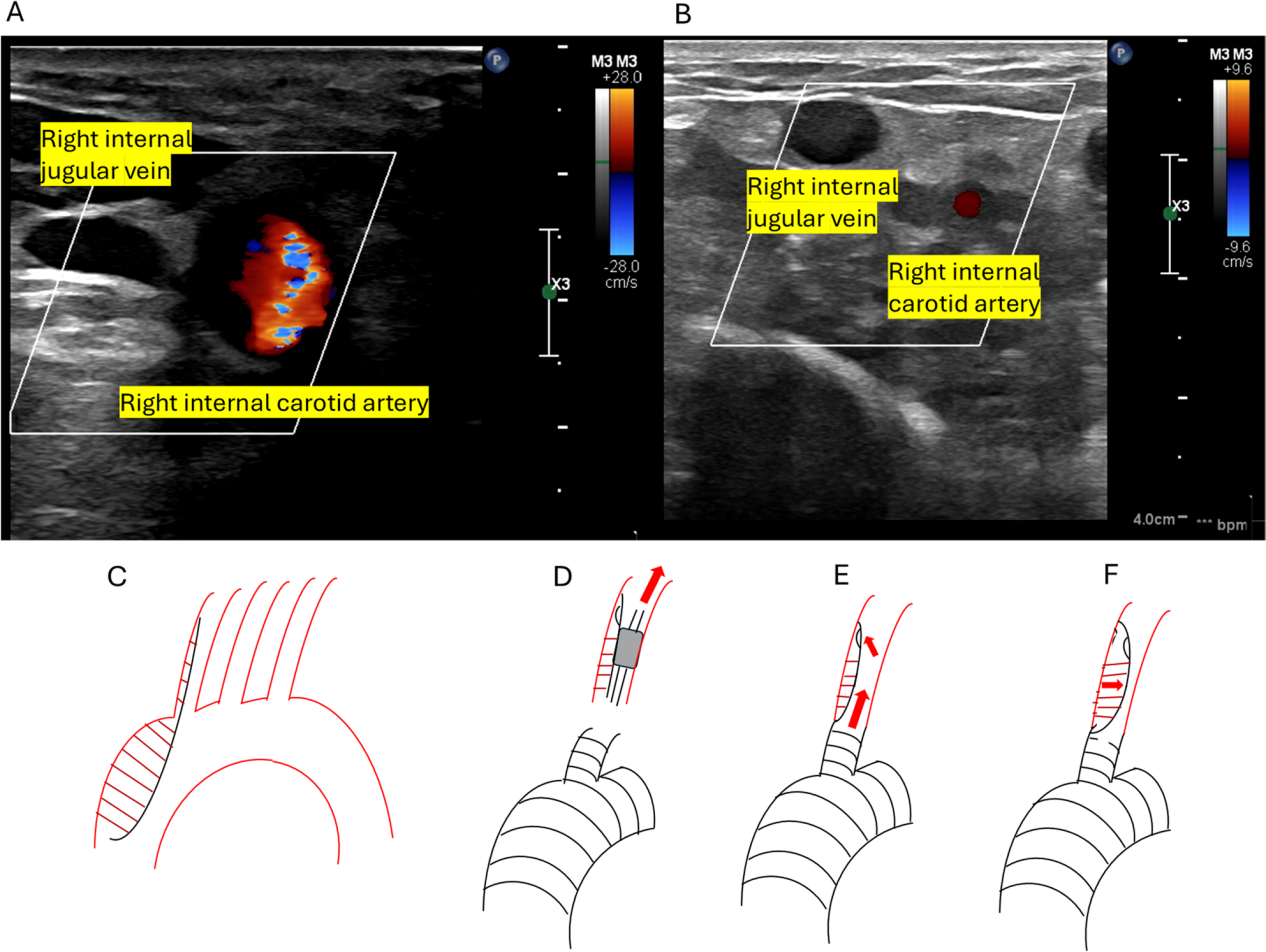
Intraoperative findings. **A** Color Doppler ultrasound of the right neck obtained with a linear high-frequency probe placed transversely at the level of the cricoid cartilage. The probe marker was oriented laterally. Color flow is observed in the right internal carotid artery, whereas venous flow is identified in the right internal jugular vein. **B** Repeat ultrasound image demonstrating markedly reduced color flow signal in the right internal carotid artery, consistent with impaired arterial perfusion. **C** Schematic illustration showing the extension of the aortic dissection prior to CPB initiation. **D** Corresponding rSO₂ trend showing maintained values during CPB. **E**, **F** Schematic illustrations demonstrating the suspected mechanism of true lumen compression caused by reperfusion from a distal reentry site, leading to false lumen expansion and decreased rSO₂

During CPB, despite ultrasonographic evidence of dissection in the cervical vessels (Epic7; Philips, Netherlands), continuous flow in the right common carotid artery was observed, and rSO₂ remained stable at 60–70% (Fig. 3A, D). Surgical findings revealed an intimal tear in the ascending aorta, with extension of the dissection into all cervical branches. The reconstruction sequence was as follows: distal aortic arch, left subclavian artery, proximal arch, LCCA, and finally, the BCA. Upon weaning from CPB, both sides of the rSO₂ suddenly decreased to 30% (Fig. 2, Event 2), accompanied by a recurrent decrease to 17 mmHg in the right superficial temporal artery pressure. Because TEE failed to depict the lesion, carotid duplex ultrasound examination using a linear probe (L12-5, Philips) demonstrated right common carotid artery collapse with expansion of the false lumen (FL), compression of the true lumen (TL), and reduced pulsatile flow (Fig. 3B). This phenomenon was suspected to result from reperfusion via a distal re-entry site, which caused the FL to compress the proximal TL upon restoration of pulsatile systemic flow, transition from the SCP, and an abrupt increase in systemic blood pressure (Fig. 3E, F). To address this, reanastomosis of the BCA was performed using an interposed graft placed more distally to exclude the reentry site. Following reanastomosis, right rSO₂ values recovered to above 60%, and the right superficial temporal artery pressure increased to 50 mmHg. Re-anastomosis of the brachiocephalic artery at a more distal site excluded the suspected distal re-entry, thereby decompressing the false lumen, relieving true lumen compression, and restoring antegrade flow to the right carotid artery.

The total operative time was 516 min, with a cardiopulmonary bypass time of 249 min and a selective cerebral perfusion time of 200 min. The total infusion volume was 14,112 mL, red blood cell transfusion volume 840 mL (6 units), fresh frozen plasma 38 units (4560) mL, and platelet concentrate 35 units. Estimated blood loss was approximately 7636 mL, and urine output during surgery was 1290 mL.

Sedation was discontinued 4 h after ICU admission on postoperative day (POD) 0; however, the patient remained at a consciousness level of Glasgow Coma Scale (GCS) E1V(T)M3. A head CT on POD 2 showed no abnormalities, whereas a repeat CT on POD 4 suggested possible hypoxic encephalopathy. Delayed awakening persisted, and on POD 5. Edaravone (60 mg/day), glycerol (400 mL/day), and nicotinic acid (1000 mg/day) were initiated. Subsequent CT scans continued to show findings suspicious for hypoxic encephalopathy. A tracheostomy was performed on POD15, and the patient was weaned from the ventilator on POD16. He was transferred to the general ward on POD20. The level of consciousness gradually improved, and he opened his eyes to verbal stimulation on POD27 (GCS E2V(T)M4). However, purposeful movements of the extremities did not recover, and by POD63 no limb movement was observed. Because of the lack of significant neurological improvement, he was transferred to a long-term care hospital.

Throughout the course, because the patient remained at GCS E1V(T)M3 or lower until late recovery, hyperactive delirium was not observed. CAM-ICU and ICDSC were not assessed due to persistent impaired consciousness. Postoperative cognitive dysfunction could not be evaluated for the same reason.

## Discussion

Several prior reports have highlighted the importance of early detection of cerebral malperfusion during cardiopulmonary bypass [2–4]. These include cases in which the drainage cannula was dislodged toward the right atrium during the Glenn procedure, resulting in inadequate venous drainage and a rapid decrease in rSO₂, as well as cases in which the arterial cannula was inserted too deeply into the brachiocephalic artery, leading to impaired perfusion of the right common carotid artery.

Intraoperative differences in rSO₂ between the left and right hemispheres have been reported to aid in the detection of cerebral malperfusion, such as in cases involving misplacement of the SCP cannula into the brachiocephalic artery and extension of aortic dissection into the carotid arteries [5, 6]. In addition, a carotid duplex ultrasound examination has been shown to be useful in detecting brachiocephalic artery malperfusion during surgery [7]. However, to our knowledge, there are no prior reports describing the combined use of rSO₂ monitoring and carotid duplex ultrasound for real-time identification of cerebral malperfusion during aortic surgery. In particular, during surgery for acute aortic dissection, intraoperative extension of the dissection may occur, and when an abrupt decrease in rSO₂ is observed, carotid duplex ultrasound should be considered as a complementary diagnostic modality to aid in identifying the cause of cerebral malperfusion.

In emergency TAR for AAD, preoperative assessment of the circle of Willis and intracranial collateral circulation is often limited, which may contribute to the risk of perioperative cerebral malperfusion. Moreover, previous studies have demonstrated that even after apparently successful central aortic repair, dissection or re-entry involving the brachiocephalic artery may persist [8, 9]. Residual false lumen pressurization caused by brachiocephalic branch re-entry has been shown to contribute to postoperative patency of the false lumen and subsequent aortic events, even in the absence of anastomotic leakage. Therefore, anesthesiologists should be aware that supra-aortic vessel malperfusion may newly manifest or worsen after restoration of pulsatile systemic flow, despite completion of arch reconstruction.

TEE is widely used for the diagnosis of acute aortic dissection and has been reported to have high sensitivity and specificity compared with transthoracic echocardiography [10]. However, given the possibility of both false-positive and false-negative findings, TEE is best used in conjunction with other diagnostic modalities to avoid misdiagnosis. Although TEE is not a continuous monitoring tool, it is utilized in response to clinical changes. Therefore, as in the present case, when a decrease in rSO₂ is observed, TEE and carotid duplex ultrasound examination using a linear probe may be useful for assessing the progression of dissection and guiding clinical decision-making. Carotid duplex ultrasound examination may be considered a diagnostic option when rSO₂ decreases.

The cause of bilateral rSO₂ desaturation remains unclear. Although false lumen expansion was confirmed only in the right common carotid artery, bilateral changes may have reflected insufficient collateral circulation and hemodynamic instability after CPB.

In acute aortic dissection, when a residual false lumen persists postoperatively in the brachiocephalic artery, the incidence of neurological complications—such as cerebral infarction or transient ischemic attack—has been reported to be approximately 30% [11]. In the present case, although a residual false lumen was identified intraoperatively, this finding was considered clinically relevant considering the previously reported postoperative risk. Nevertheless, we anesthesiologists must continue to strive for early detection of intraoperative changes in cerebral blood flow using a multimodal monitoring approach.

In conclusion, multimodal monitoring, including near-infrared spectroscopy, rSO₂ trend patterns, TEE, and carotid duplex ultrasound examination, can facilitate the detection and verification of cerebral malperfusion during AAD surgery. The integration of these modalities allows for timely modifications in surgical strategies and prompt restoration of cerebral perfusion, potentially improving neurological outcomes.

## Data Availability

Not applicable.

## Abbreviations

AAD: Acute aortic dissection
BCA: Brachiocephalic artery
CPB: Cardiopulmonary bypass
FL: False lumen
LCCA: Left common carotid artery
rSO₂: Regional cerebral oxygen saturation
TAR: Total aortic arch replacement
TEE: Transesophageal echocardiography
SCP: Selective antegrade cerebral perfusion
TL: True lumen
NIRS: Near-infrared spectroscopy
